# Editorial: Molecular mechanisms of lung endothelial permeability

**DOI:** 10.3389/fphys.2022.976873

**Published:** 2022-07-22

**Authors:** Narasimham Parinandi, Evgenia Gerasimovskaya, Alexander Verin

**Affiliations:** ^1^ Division of Pulmonary, Critical Care, and Sleep Medicine, Department of Internal Medicine, The Ohio State University Wexner Medical Center, Columbus, OH, United States; ^2^ Division of Critical Care Medicine, Department of Pediatrics, University of Colorado Denver, Aurora, CO, United States; ^3^ Vascular Biology Center and Department of Medicine, Medical College of Georgia at Augusta University, Augusta, GA, United States

**Keywords:** endothelium, cytoskeleton, lung injury, paracellular permeability, transcytosis

Lung endothelium regulates movement of fluid, macromolecules, and leukocytes into the interstitium and subsequently into the alveolar air spaces. The monolayer of endothelial cells (ECs) lining the blood vessels are in close contact and tight association with each other forming a tight barrier (Komarova and Malik, 2010; Wettschureck et al., 2019). Any breach in the endothelial barrier results in the uncontrolled movement of fluid, macromolecules and leukocytes into the interstitium and pulmonary air spaces causing pulmonary edema and inflammation (Ware and Matthay, 2000; Johnson and Matthay, 2010). Therefore, the integrity of the pulmonary EC monolayer is a critical requirement for preservation of pulmonary function. Disruption of lung endothelial barrier occurs during the inflammatory disease states such as acute lung injury (ALI) and acute respiratory distress syndrome (ARDS), which remain a major cause of morbidity and mortality with an overall mortality rate of 25%–40% (Rubenfeld et al., 2005; Maca et al., 2017). The pharmacological treatment of ALI remains non-specific and relies on supportive care and control of initiating causes (Gonzales et al., 2015; Huppert et al., 2019). Therefore, novel therapies are urgently sought after to improve the clinical outcomes. This special issue provides an overview of recent studies in the field of lung endothelial permeability with the goal to advance our knowledge of the mechanisms of pulmonary endothelial barrier regulation. We hope that it will help to identify novel strategies and pharmacologic agonists for therapeutic intervention of ALI/ARDS.

Permeability across endothelial and epithelial cell monolayers can involve transcellular and paracellular transport or both pathways (Komarova and Malik, 2010; Wettschureck et al., 2019). However, the majority of trafficking occurs through the paracellular pathway (Komarova and Malik, 2010; Wettschureck et al., 2019). Paracellular permeability is determined by equilibrium of the competing contractile forces, which generate centripetal tension *via* activation of the actomyosin contractile machinery and adhesive cell-cell and cell-matrix tethering forces, which depend upon the adhesive molecules located at cell-cell and cell-matrix contacts (Dudek and Garcia, 2001; Bogatcheva and Verin, 2008; Vandenbroucke et al., 2008). Both competing forces are linked through the actin microfilaments, which are connected to multiple membrane adhesive proteins of the zona occludens and zona adherens, glycocalyx components, functional intercellular proteins, and the focal adhesion complex proteins. Reorganization of the endothelial cytoskeleton leads to alteration of cell shape and provides a structural basis for the increase of vascular permeability (Dudek and Garcia, 2001; Bogatcheva and Verin, 2008; Vandenbroucke et al., 2008). While many edemagenic agonists like thrombin and endotoxin (lipopolysaccharide, LPS) increase endothelial permeability via the activation of EC contractility, other agonists such as phorbol esters increase EC permeability without augmenting the contraction, however, all of them weaken EC barrier through decline or reduction of the endothelial junctions and cell-matrix contacts (Garcia et al., 1995; Bogatcheva et al., 2003; Kasa et al., 2015).

The review of Karki and Birukova published in this Research Topic summarizes the current view on the paracellular mechanisms of endothelial barrier regulation focusing on the role of microtubule (MT) network. It is widely accepted that reorganization of the endothelial cytoskeleton, which is composed of actin filaments, microtubules, and intermediate filaments, provides a structural basis for vascular permeability changes (Dudek and Garcia, 2001; Bogatcheva and Verin, 2008; Vandenbroucke et al., 2008). However, despite decades of intense research, the role of microtubules in the regulation of vascular permeability remains not fully understood. Microtubules are highly dynamic cylindrical structures, composed of α,β-tubulin heterodimers, which undergo continuous assembly and disassembly (Gudimchuk and McIntosh, 2021). The assembly and stability of microtubules is regulated by 1) the nucleotide association/binding and reactivity with tubulin (binding and hydrolysis of GTP by tubulin subunits (Desai and Mitchison, 1997), 2) the interaction with cellular factors like MT-associated proteins (MAPs) (Bodakuntla et al., 2019), 3) by tubulin covalent modification like acetylation (Nekooki-Machida and Hagiwara, 2020). It was shown that microtubule depolymerization by the MT inhibitors or partial disruption of peripheral MT network by edemagenic agonists such as thrombin or tumor necrosis factor-α (TNF-α) in the lung EC is associated with dissolution of the cortical actin cytoskeleton, myosin light chain (MLC) phosphorylation, increased stress fiber formation, contraction, and EC barrier dysfunction indicating of importance of the MT-actomyosin crosstalk in the regulation of EC permeability (Verin et al., 2001; Birukova et al., 2004a; Tar et al., 2004). These effects were linked to the activation of small GTPase Rho mediated by Rho-specific guanine nucleotide exchange factor, GEF-H1, which bound to MT in the inactive state, but activates upon release from MTs. Thus, disruption of the MT network may trigger Rho-dependent contractile mechanisms leading to EC barrier dysfunction (Birukova et al., 2006). Conversely, MT stabilization by paclitaxel (taxol) or inhibition of Rho pathway attenuates or reverses endothelial hyperpermeability induced by MT disruption (Birukova et al., 2006; Birukova et al., 2010). It was also reported that other factors that maintain EC barrier such as increase in cAMP, inhibition of heat-shock protein 90 (HSP90) and p38 mitogen-activated protein kinase (p38 MAPK), activation of phosphatase 2A reveal their EC barrier protective effects, at least in part, upon stabilization of the MT network (Birukova et al., 2004b; Birukova et al., 2005; Tar et al., 2006; Antonov et al., 2008). Further, EC barrier integrity preservation is tightly linked to MT-mediated activation of small GTPase, Rac1 (Tian et al., 2012; Tian et al., 2014a), which in many cases opposes the EC barrier disruptive effects of Rho (Wojciak-Stothard and Ridley, 2002). In particular, EC barrier enhancement induced by the hepatocyte growth factor (HGF), at least in part, depends upon the activation of specific Rac1 GEF, Asef, which translocates to the EC membrane and forms a complex with the MT-binding protein, Adenomatous Polyposis Coli (APC) and Ras GTPase-activating-like adaptor protein, IQGAP, after the HGF stimulation (Tian et al., 2015a; Tian et al., 2015b). Several lines of evidence have implicated other MAPs, such as Cytoplasmic linker protein 170 (CLIP-170), End Binding Protein-1 (EB-1), stathmin, MAP4, and adaptor actin-binding protein, cortactin in the MT-mediated permeability in various models, highlighting a complex involvement of the MT dynamics in EC barrier regulation (Tian et al., 2012; Tian et al., 2014a; Tian et al., 2014b; Li et al., 2015; Karki et al., 2021a). Emerging evidence indicates that MT stability may be regulated by post-translational modifications such as acetylation (Nekooki-Machida and Hagiwara, 2020). Acetylation of tubulin at Lys-40 stabilizes the MT network (Portran et al., 2017). Conversely, deacetylation of the tubulin catalyzed by histone deacetylases (HDAC) facilitates disassembly of the MTs (Li and Yang, 2015). Recent studies have indicated that deacetylatin of tubulin by specific cytoplasmic HDAC, HDAC6, is involved in the EC barrier compromise (Karki et al., 2019). Downregulation of HDAC6 attenuates the EC barrier compromise induced by several edemagenic agents like *Staphylococcus aureus*, thrombin, LPS, and TNF-α *in vitro* and *in vivo* highlighting potential therapeutic value of HDAC6 inhibition in the treatment of ALI (Yu et al., 2016; Karki et al., 2019; Kovacs-Kasa et al., 2021). Finally, newly published studies by Karki et al. (2021a) and Karki et al. (2021b) underscore the importance of cytokine signaling/MT interaction in the regulation of EC barrier function.

While the majority of trans-endothelial trafficking of fluids and leukocytes occurs by the paracellular pathway, growing evidence has also highlighted the importance of the transcellular pathway (trancytosis) in mediating the leucocyte diapedesis and albumin transport (Komarova and Malik, 2010; Filippi, 2016). While regulating independently both the para- and trans-cellular pathways are interconnected in the regulation of tissue fluid homeostasis (Komarova and Malik, 2010). In the current Research Topic, Jones and Minshall have discussed a role of endothelial transcytosis during ALI/ARDS. Trafficking through endothelial cells happens mainly through the caveolae-dependent mechanism (Jones and Minshall, 2020). Caveolae, the lipid raft plasma membrane microdomains, enriched in the scaffolding proteins, are called “caveolins.” Caveolin 1 is required for caveola formation in the non-muscle cells including endothelium (Maniatis et al., 2012). Early immunocytochemical studies have demonstrated that transcytosis is primarily responsible for the trafficking of large molecular weight molecules such as albumin from the luminal to the basal surface of ECs (Milici et al., 1987). Animal studies have indicated fivefold increase in tracer transport associated with active trancytosis in the LPS-induced rabbit ALI model accompanied by increased abundance and internalization of caveolae, suggesting that transcytosis significantly contributes to the development of ALI (Heckel et al., 2004). Furthermore, LPS increases caveolin 1 phosphorylation *via* CD14/Src-mediated mechanism, resulting in subsequent NF-kB activation and release of pro-inflammatory cytokines suggesting direct involvement of caveolin1 activation in LPS-induced ALI (Jiao et al., 2013).

It is generally accepted that trancytosis includes three stages: 1) endocytosis, 2) vesicular trafficking, and 3) exocytosis (Simmons et al., 2019; Jones and Minshall). Recent studies have identified several key proteins involved in specific stages of transcytosis. For example, plasmalemmal vesicle-associated protein (PLVAP, PV1) apparently controls the internalization of caveolae (Jones et al., 2020). EH domain-containing protein 2 (EHD2), a member of dynamin family proteins, restricts fission and vesicle trafficking (Stoeber et al., 2012). Fractionation studies have suggested that several groups of proteins such as N-ethylmaleimide-sensitive factor (NSF), Soluble NSF Attachment Proteins (SNAPs) and SNAP receptor (SNARE) proteins are involved in the fusion of vesicles to the abluminal membrane by exocytosis of the vesicular content into the sub-endothelial space (Predescu et al., 2001; Yamakuchi et al., 2008). Similar to the paracellular permeability, transcytotic mechanisms are critically dependent upon the cytoskeletal dynamics. Electronic microscopy studies have revealed that caveolae are localized near the cortical actin filaments (Rohlich and Allison, 1976; Singer, 1979). Early studies by Shasby et al. (1982) have shown that the depolymerization of actin filaments increases albumin transport across lung ECs *in vivo* resulting in pulmonary edema. In contrast to the actin filaments, depolymerization of MTs results in an increase of the number of membrane-bound caveolar vesicles (Mundy et al., 2002), suggesting negative effect on the vesicle trafficking. In addition, caveolae recruit and activate Rho family GTPases and several other signaling molecules involved in the cytoskeletal dynamics (Ellis and Mellor, 2000; Hetmanski et al., 2019). Therefore, both para- and trans-cellular permeability pathways are ultimately involved in the cytoskeletal remodeling, resulting in the onset/development of ALI/ARDS. Thus, elucidating the mechanisms of lung endothelial transcytosis may hasten the development of new therapies towards attenuating the vascular leak associated with these debilitating pulmonary diseases.

Among other pro-inflammatory cytokines, TNF-α is a well-known mediator of inflammatory tissue damage, which plays an important role in ALI/ARDS (Malaviya et al., 2017). However, due to the TNF-α molecular complexity harboring spatially distinct domains, TNF-α can function either in the damaging or protective manner in ALI (Lucas et al.). In the review for the current Research Topic, Lucas et al. have further discussed the complex role of TNF-α domain organization and signaling in the regulation of pulmonary EC barrier and alveolar fluid clearance (AFC). Impairment of the EC barrier and AFC is a cardinal feature of ALI/ARDS (Ware and Matthay, 2000; Johnson and Matthay, 2010; Vadasz and Sznajder, 2017). TNF-α exists in both soluble and membrane-bound forms, which binds to two types of membrane-associated receptors: TNF receptor 1 (TNFR1) and 2 (TNFR2). TNFR1, but not TNFR2 contains a death domain, which can signal for either apoptosis, necroptosis or inflammation (Wajant and Siegmund, 2019). In contrast, while TNFR2 can exacerbate TNFR1-mediated apoptosis by decreasing the expression of anti-apoptotic molecules (Wajant et al., 2003), its stimulation can protect against the ventilation-induced lung injury (VILI) (Wilson et al., 2007). Collectively, while TNFR1 and 2 engage different pathways, they are interconnected in the regulation of TNF-α-mediated complex signaling network (Wajant and Siegmund, 2019). In addition to the TNFR binding sites, TNF-α carries a lectin-like domain (Yang et al., 2010), which is spatially and functionally distinct from the receptor binding domains. The lectin-like domain can be mimicked by the 17 amino acid TIP peptide (a.k.a. AP301, Solnatide) (Lucas et al.). This peptide has been shown to exert potent protective activities in several animal models of ALI (Braun et al., 2005; Hartmann et al., 2013a; Hartmann et al., 2013b). Apparently, TIP peptide-mediated protection against ALI depends upon its direct binding and activation of the epithelial sodium channel (ENaC), a key enzyme regulating AFC (Czikora et al., 2014; Lucas et al., 2016; Czikora et al., 2017). Importantly, the TIP peptide does not impair the anti-bacterial activities of TNF-α (Lucas et al., 1997) nor its receptor 1 or 2 binding capacity (Hribar et al., 1999). TIP peptide is currently evaluated in the clinical trials in ARDS and COVID-19 (Lucas et al.).

The mechanisms that govern the highly clinically relevant process of increased EC permeability are under intense investigation, however, the information about the processes that determine barrier enhancement or preservation are limited. Next three research articles in the Research Topic uncovered some new molecular mechanisms of EC barrier protection in ALI setting.

Endothelial cell-matrix tethering forces, controlled, at least in part, by the transmembrane cell adhesion receptors, called integrins, are critically involved in the endothelial barrier regulation (Malinin et al., 2012; Ou et al., 2021). In their study for the current Research Topic, Chen et al. have reported that the truncated splice variant of Integrin Beta 4, ITB4E, specifically upregulated by the HMG-CoA reductase inhibitor, simvastatin. Simvastatin is a statin family member and FDA-approved drug for lowering the level of low-density lipoprotein cholesterol in the blood (Fox et al., 2007). Published reports from the same group demonstrated barrier-protective role in EC barrier regulation (Jacobson et al., 2004; Chen et al., 2008). Ectopic expression of ITB4E in human pulmonary artery ECs (HPAECs) leads to attenuation of the LPS-induced pro-inflammatory MAPKs (ERK and JNK) activation and reduction of pro-inflammatory cytokine (IL-6 and IL-8) expression, suggesting an anti-inflammatory role of ITB4E. Further, expression of ITB4E has been shown to attenuate the HPAEC barrier compromise induced by the edemagenic agonist, thrombin. These findings implicate ITB4E expression in the endothelial barrier protection induced by statins and may facilitate the development of novel and effective pathway-specific anti-edemagenic agents that will restore endothelial function in ALI.

It is widely accepted that increase in cAMP and activation of Rac1 GTPase are involved in the preservation of EC barrier integrity (Birukov and Karki, 2018). Published reports indicate that edemagenic conditions reduce cAMP level and Rac1 expression resulting in vascular leak (Schlegel and Waschke, 2009; Xia et al., 2019). Previous studies have demonstrated that agonists of bitter taste receptors (T2Rs), a family members of G-protein-coupled receptors (GPCRs), are effective against lung inflammation in asthma (Devillier et al., 2015), however, the role of T2Rs in EC barrier regulation has not been described. The study of Kertesz et al. for the current Research Topic have demonstrated that T2Rs are expressed in HPAECs. Furthermore, the T2Rs agonists, denatonium (for T2R10) and phenylthiourea (for T2R38), attenuate the HPAEC barrier compromise via cAMP- and cAMP/Rac1-dependent mechanisms, respectively. However, phenylthiourea alone does not protect lung endothelium in the bacterial model (*P. Aeruginosa*) of ALI, therefore, barrier protective role of T2Rs *in vivo* requires further investigation.

Recently published data (Beumer et al., 2019; Chen et al., 2020) suggests that combined bacterial infection in the lung exacerbates ARDS and multiple organ failure induced by COVID-19 infection. COVID-19-induced vascular inflammation and compromise of the EC function relies on the binding of surface spike glycoprotein (S protein, SP) to angiotensin-converting enzyme 2 (ACE2) in the endothelium (Lei et al., 2021). S protein comprised of two functional subunits, namely S1 and S2, activated by protease cleavage, mediate attachment and membrane fusion, respectively (Banerjee et al., 2022). Published literature indicates that S protein alone can damage EC *in vitro* and *in vivo* (Lei et al., 2021). Recent report from Catravas’s group (Colunga Biancatelli et al., 2021) has demonstrated that cleaved S1 (S1SP) is capable to compromise human lung microvascular ECs (HMVECs) barrier function and induce ALI in mice. In their article (Colunga Biancatelli et al.) for the current Research Topic, this group further has evaluated the mechanisms of S1SP-mediated HMVEC barrier dysfunction with a focus on the protective action of inhibition of HSP90. HSP90 belongs to the family of chaperons, which assists with protein folding and stabilization (Schopf et al., 2017). Previous studies have demonstrated that HSP90 inhibitors protect and restore the EC barrier integrity *in vitro* and *in vivo* (Antonov et al., 2008; Chatterjee et al., 2008). Studies reported by Colunga Biancatelli et al. have revealed that the protective effects of HSP90 inhibitors are attributed to the attenuation of activation of IKBα and protein kinase B (PKB, AKT) induced by S1SP and accompanied by restoration of VE cadherin expression. While additional studies are required on the effect of HSP90 inhibitors in the *in vivo* models of COVID-19, the results from this current study may pave the way to the novel approach for treatment of COVID-19-associated ALI.
